# The living will system in mainland China: legislative status quo, dilemmas and prospects

**DOI:** 10.3389/fpubh.2026.1767356

**Published:** 2026-02-27

**Authors:** Longmei Tian

**Affiliations:** School of Law, Tongji University, Shanghai, China

**Keywords:** China, end-of-life decision-making, legislation, living will, patient autonomy

## Abstract

As the right of patients to make autonomous decisions about life-sustaining treatment has gained wider recognition, many countries have introduced legislation on living wills. Over the past two decades, mainland China has seen a gradual shift from voluntary advocacy by civil-society organizations to initial local legislation, culminating in the Shenzhen Special Economic Zone Medical Regulations (2023), the first statute to recognize the legal effect of living wills. Yet the emerging living will system continues to be hindered by multiple factors at both the legislative and implementation stages. This article identifies three main challenges: vague statutory definitions of key concepts; the lack of supporting mechanisms, particularly for registration and revocation; and tensions between the practice of living wills and traditional cultural understandings of death and filial piety. On this basis, it proposes reforms to clarify in legislation the qualifications of parties eligible to execute living wills and the scope of their application, to establish a unified and accessible registration–revocation system, and to reinterpret the normative foundations of the living will system in light of local cultural values. The analysis aims to provide a roadmap for strengthening institutional safeguards for patients' medical autonomy in China and to offer comparative insights for other developing countries considering similar legislation.

## 1 Introduction

The concept of the living will was first proposed in 1969 by Kutner, who argued that since property law permits individuals, while mentally competent, to make advance dispositions concerning the distribution of their assets, they should likewise be entitled to formulate and sign advance instructions regarding end-of-life medical decisions (1). As a result, the living will gradually become a subject of public debate and academic research. From the 1970s onward, European countries, the United States and other jurisdictions began to explore the living will system, gradually developing a comprehensive institutional framework and practical pathways. In 1976, California took the lead in the United States by enacting the *Natural Death Act* (NDA), which legally vested patients with the right to choose a natural death; thereafter, other states successively adopted similar legislation. In 1990, the United States Congress enacted the *Patient Self-Determination Act* (PSDA), which conferred statutory recognition on living wills and granted patients the right to accept or refuse medical and surgical treatment. In 1993, the Uniform Law Commission (ULC) adopted the *Uniform Health-Care Decisions Act* (UHCDA), which set out detailed provisions governing living wills. Subsequently, building on the objectives of the original act, the ULC modernized and expanded the UHCDA and approved the *Uniform Health-Care Decisions Act (2023)* (2), thereby further enhancing the practical applicability of living wills. Nearly 50 years of legislative practice have made the United States the earliest country in the world both to enact legislation on living wills and to implement such legislation effectively (3). Thereafter, countries such as the United Kingdom, Germany, Canada, South Korea and Singapore, as well as regions including Taiwan and Hong Kong of China, also enacted similar regulations establishing living will systems. The United Nations *Universal Declaration on Bioethics and Human Rights* further reaffirms individual autonomy from a human rights perspective, emphasizing that special measures should be taken to protect the interests of those who lack the capacity to exercise their autonomy (4). The World Health Organization's recently issued *Patient Safety Rights Charter* likewise reiterates and underscores patients' right to dignity—particularly their dignity and autonomy in sensitive contexts such as palliative care and the end of life (5).

In 2016, the *Outline of the “Healthy China 2030” Plan* explicitly set forth the core objective of “maintaining and safeguarding the people's health in an all-round manner and throughout the entire life course” (6). Guided by this life-course health concept, issues concerning quality of life and dignity at the end of life have gradually attracted broad attention across society, creating an opportunity for the establishment of a living will system. At the same time, China's increasingly severe population aging has generated an urgent practical need to further promote living wills. Statistics show that in 2024 the number of people aged 60 and above in mainland China reached 296 million, accounting for 21.1% of the total population; it is projected that around 2050 the older population will peak at 487 million, or 34.9% of the total population, among whom those aged 80 and above will reach 150 million (7). Against the backdrop of a continuously deepening degree of population aging, the living will system has become an important legal instrument for China to respond to the challenges of end-of-life medical care in an aging society, to implement palliative and hospice services, and to safeguard citizens' autonomy at the terminal stage of life.

At present, Chinese laws and regulations have not yet clearly defined the concept of a living will; according to the prevailing view in academia, a living will refers to an instructional document signed when an individual is mentally competent, indicating whether, and what kind of, medical and nursing care should be provided when the person is in the terminal stage of an incurable illness or at the end of life (8).

From 2006, when the concept of living wills was first introduced into mainland China, to 2023, when Article 78 of the *Shenzhen Special Economic Zone Medical Regulations* (the Shenzhen Regulations) for the first time established a living will system in legislation, China's living will system has undergone continuous development and refinement. Nonetheless, it should be made clear that this system in China is still at an early stage, and both legislation and implementation face many pressing problems, such as the absence of unified and clear normative provisions, the lack of consensus on key concepts, and the underdevelopment of supporting implementation mechanisms. Compared with society's urgent demand for living wills under the trend of population aging, the current pace of institutional development is clearly lagging, and the urgency of improving legislation on living wills and optimizing their implementation is becoming increasingly salient.

Against this backdrop, this article aims to systematically sort out the development and current status of China's living will system, summarize the legislative gaps and practical difficulties in its implementation, and ultimately put forward targeted proposals for improving the legislation as well as preliminary ideas for constructing relevant supporting mechanisms, so as to provide a reference for promoting the standardized and law-based development of living will systems in China and other developing countries.

## 2 From social advocacy to local legislation: the evolution of living wills in China

### 2.1 Civic-society advocacy and early practice diffusion

The development of living wills in China started relatively late and has been characterized mainly by two transitions: first, at the institutional level, a shift from spontaneous advocacy by civic associations to official exploration of legislative practice. In 2006, drawing on the experience of the United States, the concept of the “living will” was introduced into mainland China for the first time through the website Choice and Dignity. In 2013, the first living will promotion association in mainland China was established in Beijing; building on the US. living will template Five Wishes and taking into account China's national context, the association introduced a living will document entitled My Five Wishes for use by mainland residents, which, in the form of a questionnaire, guides individuals in arranging end-of-life medical measures in accordance with their wishes and treatment preferences. In 2015, the China Association for Life Care released the *China Living Will Promotion Plan*, marking the entry of living wills in mainland China into a phase of substantive promotion. Beijing, Shanghai, Guangdong, Jiangsu, Zhejiang and other provinces have since carried out practical explorations of living wills. Second, at the societal level, attitudes have shifted from passive observation to active acceptance. Three surveys conducted by the Beijing Living Will Promotion Association between 2006 and 2015 indicate that public awareness of, and willingness to complete, living wills have risen year by year (9), and by 2022 a total of 57,187 people in China had registered their living wills (10). Since the Shenzhen Regulations incorporated living wills into legislation, related practice has expanded rapidly. Attorney Du Qin executed a living will for herself at the Shenzhen Notary Office, which became the first notarized living will in Shenzhen after the law was enacted (11). As of March 2024, the Shenzhen living will registry had recorded 33,084 entries by March 2024, with registrants displaying a pronounced trend toward younger age groups, and related topics have sparked extensive discussion across the country (12).

These developments indicate that, over the past two decades, China's living will system has undergone a gradual yet substantive transition from grassroots advocacy to institutional practice, accompanied by a steady rise in societal awareness and acceptance of autonomy at the end of life. Nevertheless, against the backdrop of China's increasingly acute population aging, a pronounced mismatch persists between the existing institutional supply of living wills and the practical needs of an aging society. The wave of population aging has not only exacerbated the imbalance between the supply of and demand for medical resources, but has also generated an urgent need among large numbers of functionally dependent and partially dependent older adults for support in end-of-life medical decision-making. Owing to illness, many such older adults may experience impaired consciousness or lose the capacity to communicate; in the absence of an advance specification of their treatment preferences through a living will, they risk being caught in a dilemma between “overtreatment” and “undertreatment,” while simultaneously placing substantial caregiving burdens and ethical decision-making pressures on their families. Driven by this practical context, the evolution of China's living will system is no longer confined to latent, spontaneously expressed demands at the societal level, but has increasingly taken the form of an explicit, time-sensitive claim for legal safeguards at the national level. Only the legislative establishment of a unified, clearly articulated and practicable institutional framework can move living wills from being a discretionary option for a limited group of individuals to a mainstream mechanism for protecting the rights of the majority.

### 2.2 National policy support and Shenzhen local legislative breakthrough

China has long relied on an incremental legislative model characterized by “pilot projects first, then scaling up”, and the legislative evolution of the living will system is no exception. The development of China's living will system can be divided into three stages (Supplementary Table 1).

The first is the stage led by policy documents and legislative initiatives. In this stage, the State Council's issuance of dedicated opinions, together with legislative proposals submitted by deputies to the National People's Congress (NPC) and members of the Chinese People's Political Consultative Conference (CPPCC), have served as the main vehicles for clarifying the direction of reform and the need for legislation, thereby providing a value basis and policy foundation for subsequent law-making. In 2013, the State Council issued the *Several Opinions on Promoting the Development of the Health Service Industry*, which encouraged the development of and improvement in the quality of palliative care services, thus supplying policy support for the promotion of the living will system (13). As a key practical vehicle for living wills, palliative care centers on respecting patients' end-of-life medical preferences and safeguarding life dignity, and therefore closely corresponds to the core meaning of living wills—namely, the ex-ante specification of end-of-life treatment choices. In the same year, many CPPCC members and NPC deputies submitted proposals advocating the promotion of a living will system. Thereafter, proposals concerning living will legislation have been submitted by NPC deputies or CPPCC members almost every year (14). Article 8 of the 2017 expert draft of the *Personality Rights Book of the Civil Code* asserted that natural persons have the right autonomously to decide to forgo medical treatment, thereby directly embodying the core connotation of living wills. Although the final text of the *Civil Code* did not directly adopt this opinion, Article 1,002 on the “right to life” explicitly provides that “the safety and dignity of the life of a natural person are protected by law,” thereby incorporating “life dignity” into the catalog of statutory rights. Some scholars have observed that “the core of life dignity lies in dignity in death” (15), and that dignity in death depends to a considerable extent on the quality of life in the terminal phase. Living wills, as a concrete institutional arrangement for specifying end-of-life medical preferences in advance and enhancing the quality of life at the end of life so as to safeguard life dignity, therefore find their principal doctrinal basis in Article 1,002 of the *Civil Code*. In 2019, the National Health Commission, together with other departments, jointly issued the *Guiding Opinions on Establishing and Improving the Health Service System for the Older adults*, which further provided policy support for the practical implementation of the living will system in terms of service standards, model design and public education (16).

The second is the stage of local legislative exploration. Guided by the national policy framework, certain provinces or special economic zones have undertaken legislative experiments on living wills through local regulations, thereby accumulating practical experience for national-level legislation. On 1 January 2023, the revised Shenzhen Regulations entered into force. Article 78 of these Regulations, for the first time in China, conferred legal effect on living wills: under this provision, decisions regarding emergency resuscitation at the end of life are no longer made by family members but by patients themselves (17). In addition, the Regulations specify the conditions for effectiveness, core content and forms of execution of living wills. With respect to the conditions for effectiveness, the Regulations expressly limit them to circumstances in which “the patient is in the terminal stage of an incurable injury or illness or at the end of life.” As to content, a person making a living will may choose among three categories of medical measures that are expressly recognized in the legislation: invasive resuscitation measures, life-support systems, and continuing treatment measures for the primary disease. At the same time, the inclusion of the term “etc.” leaves room for expanding the scope of medical measures in future legislation. Regarding the form of execution, the Regulations provide three modes for making a living will, namely a notarization mode, a witnessed-execution mode and an audio–video recording mode. They also impose a special restriction on witnesses: “medical and health personnel involved in the patient's treatment” are excluded from serving as witnesses, and only third parties with the capacity to act as witnesses may do so. As a breakthrough in local legislation, these Regulations directly transformed “living wills” from a policy concept into a legal norm, thereby making Shenzhen the first city in mainland China to enact specific legislation on living wills. The institutional design of the Shenzhen Regulations offers a practical model for the nationwide promotion and legislation of living wills. Following the entry into force of these Regulations, other provinces likewise initiated legislative efforts concerning living wills. Members of local political advisory bodies submitted proposals for enacting local regulations on living wills, recommending that Shenzhen's legislative experience be taken as a reference (18). Some provinces have already incorporated living wills into the legislative plans of their provincial people's congresses (19).

The third is the stage of systematic national legislation, which aims to distill the institutionally mature arrangements tested in local practice and elevate them into laws or administrative regulations of uniform application nationwide. At present, the development of the living will system in China has not yet progressed to this stage. However, in light of both the internal logic of institutional evolution and pressing practical needs, enacting unified national legislation on living wills is an inevitable choice for China in responding to population aging, optimizing the allocation of medical resources and safeguarding citizens' autonomy.

## 3 Dilemmas in the legislation and implementation of China's living will system

Although the living will system has been incorporated into the legal framework, inherent limitations of the legislation itself, together with incomplete supporting measures, mean that the application and enforcement of the law continue to face a range of difficulties. These challenges, which can be clearly categorized into deficiencies at the legislative level and obstacles at the implementation level, significantly constrain the effectiveness of living will legislation and hinder the smooth operation of the system in practice.

### 3.1 Legislative deficiencies in applicability standards and liability rules

First, as the core legal instrument through which people exercise autonomy over end-of-life medical interventions, a living will depends for its effectiveness on the standard applied to the legal capacity of the person executing it. This standard directly determines whether the end-of-life medical decisions contained in the living will can be validly implemented. Where a patient lacks the requisite capacity at the time of making a living will, the will is rendered invalid. It is therefore necessary to clarify who is qualified to make a living will, that is, to specify what level of legal capacity a person must have for the will to be valid. However, the Shenzhen Regulations merely provide that a living will may be submitted to a medical institution by “the patient or their close relatives,” without explicitly stipulating the capacity requirements for the person executing the will. On the question of how to assess a patient's capacity, many jurisdictions have introduced the specific concept of “medical decision-making capacity” in their medical legislation to determine whether an individual is competent to make autonomous end-of-life treatment decisions. By contrast, the current Chinese legal system has not yet established such a specialized concept. Assessment of capacity must therefore still rely on the general rules in China's *Civil Code*. Under the *Civil Code*, possession of the corresponding civil capacity is one of the necessary conditions for a civil subject's juridical acts to take legal effect. Scholars generally consider a living will to be a new type of civil juridical act unilaterally performed by a civil subject through a declaration of intention (20). Accordingly, the person executing a living will should have the corresponding civil capacity (21). Yet the classification of civil capacity is fundamentally based on a natural person's age, intellectual development and mental health status. Only living wills executed by persons with full civil capacity have legal effect under civil law (22). Minors and persons with mental disorders who have no or only limited civil capacity, even if they are to some extent able to understand and express their wishes regarding their own medical affairs, are still unable to execute a living will.

Second, the Shenzhen Regulations limit the circumstances in which a living will may take effect to two core situations: where the patient is in the “terminal stage of an incurable illness” or in a “dying state.” A living will may be implemented only when the patient is in one of these two conditions. The Shenzhen Regulations, however, do not clearly define the specific meaning of “terminal stage of an incurable illness” or “dying state,” nor do they specify who is authorized to make such determinations.

In fact, the criteria for determining the “terminal stage of illness” differ across national and regional legal systems. Singapore's *Advance Medical Directive Act* defines it as a situation in which “terminal illness means an incurable condition caused by injury or disease from which there is no reasonable prospect of a temporary or permanent recovery” (23); the United States *Uniform Rights of the Terminally Ill Act* defines a “terminal condition” as “an incurable or irreversible condition that, without the administration of life-sustaining treatment, will result in death within a relatively short time” (24). These definitions illustrate that there is already a divergence between broader and narrower understandings of “terminal” in foreign legislative practice, while Chinese legislation has not provided a precise textual definition of this concept. Moreover, Article 78 does not specify which institutions or actors are competent to assess whether a patient is indeed in the terminal stage of an incurable illness.

Such conceptual vagueness not only exacerbates confusion between patients and medical staff and among medical institutions, but may also lead to inconsistent thresholds for when a living will takes effect, thereby directly undermining the uniformity and fairness of the system's implementation.

In addition, neither Article 78 of the Shenzhen Regulations nor the chapter on legal liability establishes specific provisions on liability tailored to the living will system. More specifically, the current legislation is deficient in two respects. First, where a medical institution fails to implement a patient's living will, the existing provisions neither clarify the type of liability to be borne (for example, administrative or civil liability) nor delineate the concrete content and scope of such liability. Consequently, the requirement in Article 78 that “the expression of intent in a patient's living will shall be respected” operates only as a general, principle-based guideline for medical institutions: it merely calls upon them to attach importance to patients' expressed wishes, without conferring coercive force on the provision or imposing a binding obligation on medical institutions to provide diagnosis and treatment strictly in accordance with the living will. This absence of clearly articulated legal liability means that, within the framework of the Shenzhen Regulations, living wills lack robust, mandatory safeguards and their legal effect remains inherently limited. Second, the Shenzhen Regulations do not establish any safe-harbor or immunity provision shielding medical personnel from liability when they act in good faith in implementing a living will. Even where a living will be valid, medical personnel who are confronted with opposition from the patient's family often find it difficult to follow the patient's own wishes because they fear incurring civil, or even criminal, liability. In practice, such legal gaps can impede implementation: a survey on the implementation of living wills in Shenzhen indicates that even with a valid living will, if the patient did not communicate it to family members in advance or the family disagrees, hospitals often avoid relying on the directive alone and proceed only after relatives sign the necessary consent forms to avert disputes and litigation (25).

### 3.2 Inadequate supporting implementation mechanisms

In jurisdictions that recognize living wills, most require a unified system for the registration and filing of such instruments, which is a critical precondition for their effective implementation. Although Article 78(2) of the Shenzhen Regulations specifies three lawful forms for creating a living will—namely notarization, attestation by witnesses, and audio-visual recording—China has not yet established a nationwide, unified registration and filing mechanism for living wills. In practice, when a patient executes a living will in one of these forms before losing decision-making capacity but does not inform close relatives or medical institutions in a timely manner, the relevant parties are likely to remain unaware of the document, thereby undermining its practical effect. Even when a living will is notarized and the notary office maintains an internal record, privacy-protection requirements impose strict limits on who may access that record and under what conditions. As a result, medical institutions often cannot obtain information about a declarant's living will at the time critical treatment decisions are made, which may again result in the frustration of the declarant's genuine wishes in individual cases.

Moreover, the core value of living wills lies in fully respecting patients' decisional autonomy in medical care, and such autonomy should be reflected not only at the stage of execution but also throughout the entire process of amendment and revocation. When changes in a patient's clinical condition, understanding, or preferences lead the patient to wish to withdraw or revise an existing living will, the principle of self-determination requires that the law permit the patient to exercise the right to amend or revoke that instrument. While the Shenzhen Regulations explicitly require that living wills be created through specified formal mechanisms, such as notarization or witnessing, to safeguard the authenticity and accuracy of their contents and to prevent forgery or tampering, they do not specify the procedures by which a living will may subsequently be amended or revoked. This gap has given rise to a central practical controversy: must amendments to or revocations of a living will satisfy exactly the same formal requirements as its initial execution? For example, if a living will was executed by notarization, must any subsequent amendment also be notarized? If it was executed through witnessing, must the original witnesses appear again, or may new witnesses be engaged for any modification? If amendments and revocations are required to comply with procedures that are as stringent as those governing execution, this may create substantial practical barriers for patients with limited mobility or severe illness, thereby paradoxically constraining their autonomy. Conversely, if the formal requirements are relaxed, the validity of living wills may become more vulnerable to challenge and to malicious interference by others, making it difficult to strike an appropriate balance between procedural rigor and practical feasibility.

### 3.3 Deep-seated cultural and ethical implementation constraints

In the context of traditional Chinese culture, death has long been a highly sensitive topic. People are generally reluctant to refer to it directly and instead rely on euphemistic expressions in everyday life. The well-known Confucian dictum, “You do not yet understand life—how could you possibly understand death?” (26), has exerted a profound influence on popular thinking. This emphasis on life coupled with the avoidance of death directly constrains public acceptance of living wills. Because of limited awareness, many patients conflate living wills with euthanasia or testamentary wills and therefore misinterpret the act of signing a living will as “abandoning life” or “passively awaiting death” (27). As a result, they resist engaging with this mechanism at a psychological level and, even when confronted with terminal illness, are reluctant to consider relevant arrangements. According to a 2020 study by Luo Yuping and colleagues, the “Choice and Dignity” website—the first platform in mainland China dedicated to promoting living wills—had registered only around 50,000 users, of whom 21,618 had actually completed a living will form (28), a vanishingly small number in a country of 1.4 billion people. By contrast, survey data from the United States indicate that, among 7,946 respondents, 26.3% reported having signed an advance directive (29). This stark cross-national contrast in prevalence further highlights the cognitive barriers faced by the Chinese public.

An even more deep-seated source of resistance lies in the entrenched Confucian ideal of filial piety. The *Analects* (*Lunyu*) states that “filial piety and brotherly respect are the root of humaneness” (30), treating filial devotion as the core of moral virtue and requiring individuals to take filial piety as a basic norm in managing family and social relationships. With respect to the treatment of one's parents, the *Analects* further explains that “while they are alive, serve them with ritual; when they die, bury them with ritual and sacrifice to them with ritual” (26), emphasizing that filial obligations must be fulfilled in accordance with ritual both during parents' lifetime and after their death. Under the influence of this ethical framework, family members generally believe that, as long as the patient has not completely lost vital signs, every possible effort should be made to pursue active life-sustaining treatment, which is regarded as the direct embodiment of filial duty. Conversely, raising issues of death while the patient is still alive, or withdrawing or withholding futile treatment in accordance with a living will, is easily stigmatized as “unfilial.” This not only imposes a heavy moral burden of self-reproach on family members but may also expose them to social pressure and criticism from relatives and neighbors, thereby reinforcing instinctive resistance to the implementation of living wills.

Beyond patients and families, the cultural and ethical tensions surrounding living wills also involve physicians. In Chinese traditional medical ethics, physicians are widely regarded as morally esteemed professionals charged with “relieving the suffering of the world and saving lives” (*ji shi jiu ren*) (31). Accordingly, clinicians asked to implement a living will may experience not only liability concerns but also conscientious and moral distress. As one recent discussion of living wills notes, physicians may claim conscientious objection to implementing a living will, even when the family supports the patient's expressed wishes (32, 33).

## 4 Recommendations for addressing the legislative and implementation challenges of living wills in China

To address the legislative and implementation challenges associated with living wills, reforms must advance in three interrelated phases: further specification of statutory provisions, improvement of supporting mechanisms, and clarification of the underlying normative values of the regime.

### 4.1 Short-term: clarifying key legal concepts and liability rules in the legislative revisions

#### 4.1.1 Defining the eligibility of persons who may execute a living will

In Chinese legal scholarship, there is ongoing debate regarding who should be eligible to execute a living will. One group of scholars maintains that eligibility should be confined to persons with full civil capacity, on the grounds that such capacity presupposes an ability to adequately understand and assess both the risks associated with relevant medical interventions and the legal consequences of making a living will. By contrast, minors and adults who cannot fully recognize the nature of their own conduct may, owing to limited life experience or cognitive impairment, find it difficult to accurately appreciate the significance of medical decision-making and its potential risks (22). Other scholars, however, argue that eligibility to make a living will should be determined on the basis of decisional capacity rather than civil capacity. They point out that some seriously ill minors may possess a level of maturity exceeding that of healthy peers of the same age, and that drawing the line solely with reference to civil capacity would deprive this group of autonomy in end-of-life medical decision-making. Accordingly, they advocate the establishment of a concept of medical decision-making capacity that is independent of general capacity to act, so as to safeguard the end-of-life autonomy of minors and persons with mental disorders (34).

Although the proposal to recognize medical decision-making capacity as a concept distinct from general capacity to act is normatively appealing, it faces significant practical obstacles at the initial stage of building a living will system: Chinese law has not yet defined medical decision-making capacity or set out criteria for its assessment, and the evaluation of such capacity requires specialized medical expertise, with no consensus in the medical community on uniform assessment standards. In the absence of a nationally recognized assessment framework, an age-linked civil-capacity threshold offers a more uniform proxy for eligibility determinations—not because age perfectly tracks decisional capacity, but because developmental and clinical ethics scholarship indicates that adolescent decision-making often exhibits heightened variability in risk appraisal and future-oriented reasoning, and is more susceptible to coercion or undue influence in high-stakes contexts such as severe pain and family conflict (35). A clear, readily verifiable threshold therefore improves predictability and consistency across institutions, while reducing the risk that vulnerable persons' directives are shaped by misunderstanding, coercion, or misuse, thereby mitigating opportunities for abuse at the regime's formative stage. Moreover, comparative legislative practice suggests that many living-will regimes adopt a cautious approach in their early phase, typically limiting eligibility to “adults with full civil capacity”. For example, California's 1976 *Natural Death Act* requires the declarant to be at least 18 years old and to possess full legal capacity (36); the United Kingdom's *Mental Capacity Act* 2005, when codifying the Advance Decision to Refuse Treatment (ADRT), stipulates that the maker must be over 18 and have mental capacity (37); and Taiwan's 2015 *Patient Right to Autonomy Act* requires that the individual concerned be at least 20 years old and have full capacity to act (38). These examples all point to the same legislative orientation.

Nevertheless, this “full civil capacity” threshold should be understood as a pragmatic compromise. It may exclude mature minors who may possess sufficient understanding and voluntariness for certain end-of-life decisions, and it may also exclude individuals with early-stage dementia or fluctuating cognitive impairment who still retain decision-specific capacity for refusing particular treatments. This limitation should be acknowledged explicitly as the cost of prioritizing feasibility and protection in the system's early phase. Comparative experience illustrates that some jurisdictions attempt to accommodate this nuance through more individualized standards. In the UK, while statutory ADRT is generally limited to adults, the broader concept of Gillick competence recognizes that a minor may consent to medical treatment if he or she demonstrates sufficient maturity and understanding of the proposed intervention (39). In Canada, several provinces and the case law on the “mature minor” doctrine similarly reflect a capacity-oriented approach, emphasizing the child's actual ability to understand and appreciate consequences rather than age alone (40). These models, however, presuppose relatively mature clinical and legal infrastructure. Given that China's living will system is still in its infancy, it should develop gradually within the existing legal framework. A prudent approach is therefore to temporarily align eligibility with the Civil Code's criteria for persons with full civil capacity, while reserving a future reform pathway to introduce a decision-specific “medical decision-making capacity” standard once the necessary definitional, clinical, and procedural preconditions are established.

#### 4.1.2 Clarifying the circumstances under which living wills become effective

As noted above, the Shenzhen Regulations state only in general terms that a living will becomes effective when the patient is “in the terminal stage of an incurable injury or illness or at the end of life,” without specifying how “incurability” should be determined, what situations qualify as the “terminal stage” or “end-of-life,” or which actors are authorized to make these determinations. Although such broadly framed provisions provide flexibility in the early stages of legislation, they suffer from the drawback of indeterminacy.

In future, when adopting dedicated legislation on living wills or refining existing regulations, the conditions for effectiveness could be moderately typified by reference to common scenarios in clinical practice, thereby combining general clauses with enumerated categories. For example, informed by clinical practice, “incurable conditions” may be broadly grouped into five categories: patients with advanced-stage cancer, patients with persistent vegetative state, patients in irreversible coma, patients with multiple organ failure and no prospect of recovery, and patients with extremely severe burns (41). These categories may serve as reference points when specifying the circumstances under which a living will becomes effective. At the same time, an open-ended category such as “other comparable circumstances” could be retained to preserve the necessary degree of flexibility.

In addition to defining substantive criteria for effectiveness, the law must also clarify who is responsible for determining whether those criteria are met, as this is likewise crucial for the protection of patients' rights. Because treating physicians are most familiar with the patient's clinical condition, many jurisdictions adopt a model in which the attending physician responsible for the current course of treatment, together with a second physician or a relevant multidisciplinary team, makes the medical determination as to whether the patient has, in the legal sense, entered the terminal (end-of-life) stage. If, after conducting independent assessments, both the attending physician and the relevant specialist conclude that the patient is in the terminal stage, the requirements for the living will to take effect may be deemed to have been met. If their assessments diverge, the attending physician will typically refer the case to a clinical ethics committee, which then undertakes further review and issues recommendations on how the case should be managed.

#### 4.1.3 Clarifying legal liability and good-faith immunity in living will implementation

If the legal effect of living wills is recognized without corresponding provisions on liability, medical personnel may disregard the declarant's genuine treatment preferences at will, thereby reducing the regime to a mere formality and undermining its purpose of safeguarding dignity at the end of life. To ensure the effective implementation of living wills, appropriate legal consequences should be imposed on violations. Where medical personnel, knowing that a patient has lawfully executed a living will refusing life-sustaining or invasive treatment and that the will has become effective, nonetheless provide treatment in contravention of the patient's wishes, they infringe the patient's right to medical self-determination and personal dignity. In such cases, the patient or close relatives may claim compensation for pecuniary loss from the medical institution and seek damages for mental or emotional harm. Conversely, if medical personnel, knowing that the living will expresses the patient's wish to continue life-sustaining treatment, unilaterally terminate treatment and thereby cause the patient's death, they may be suspected of intentional homicide and incur both civil and criminal liability. At the same time, the design of liability rules must also take into account the inherent uncertainty of medical practice and the professional duty of healthcare personnel to preserve life. By establishing reasonable grounds for exemption from liability, the law should strike a balance between protecting patients' rights and safeguarding the legitimate interests of medical personnel, thereby enhancing the enforceability of living wills and reducing medical disputes.

To alleviate the fears that deter medical personnel from implementing living wills in the face of family opposition, legislation should also provide a clear “safe harbor” for physicians and institutions who, in good faith, follow a valid and applicable living will even when family members demand continued life-sustaining treatment. A workable design is to grant immunity from civil, criminal, and disciplinary liability where clinicians take reasonable steps to verify validity and applicability (e.g., identity confirmation, registry check, documentation, and an internal second opinion or ethics consultation when feasible) and then act in accordance with generally accepted clinical standards. Such good-faith immunity provisions are common in mature advance-directive regimes and directly reduce defensive medicine and non-compliance driven by fear of family retaliation (42–44).

### 4.2 Mid-term: improving the mechanisms for registration and revocation of living wills

#### 4.2.1 Establishing a nationally unified management system for living wills

China has not yet developed a unified platform for querying living will information, nor does it provide a formal channel through which medical institutions can lawfully and conveniently access such information about patients. By comparison, while the United States has not created a nationwide registry of living wills at the federal level, many states have established state-level registration systems, supplemented by privately operated national electronic registries that allow healthcare professionals to retrieve relevant directives in emergency situations. In the United Kingdom, the system relies primarily on the National Health Service (NHS): advance decisions to refuse treatment (ADRTs) made during a person's lifetime are usually submitted in writing to the general practitioner (GP), who records them in the electronic medical record so that clinicians within the NHS can consult the information when the patient presents for care. In Taiwan, living wills are recorded on the National Health Insurance card, and before such notation is made the healthcare institution must first scan the document and store the electronic file in the database of the central competent authority. In mainland China, some third-party registration initiatives have also emerged, such as the Beijing Living Will Registration Center and the China Wills Bank's “Anxin Living Will” program. However, these arrangements remain highly localized and face persistent problems of limited interoperability, weak integration with clinical workflows, and insufficient regulatory oversight and safeguards. To address these shortcomings, this article proposes that the National Health Commission take the lead in establishing a nationally unified living will management platform.

**Figure 1:**
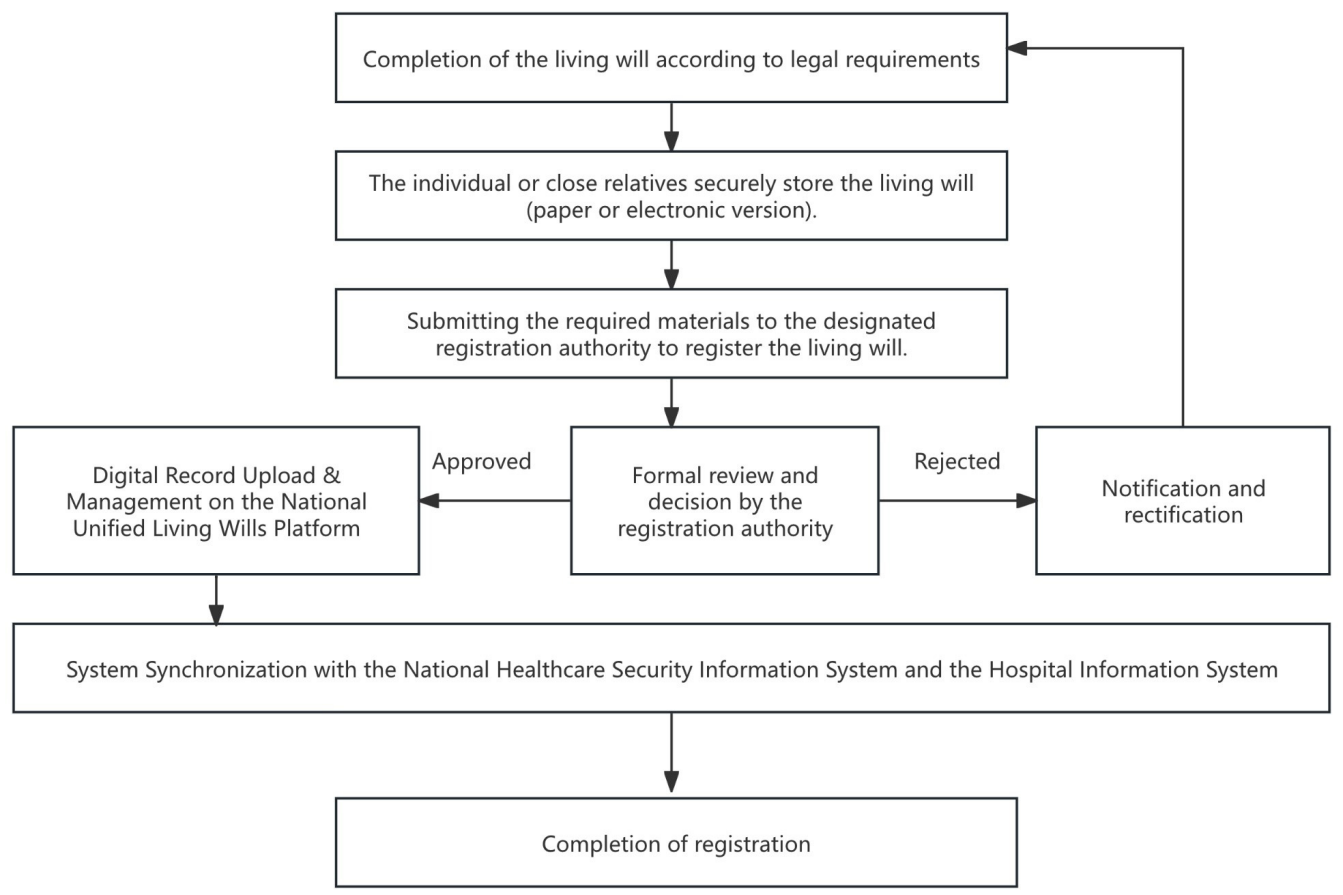
Registration process of living wills. This flowchart illustrates the standard workflow through which an individual completes and registers a legally valid living will: drafting the document while having full civil capacity, obtaining notarization or witnessing, submitting the living will and supporting materials to the designated registration body, and having the information entered into a unified management platform that can be accessed by authorized medical institutions.

On this basis, the following institutional design can be envisaged (Figure 1). Once a living will has been duly executed in accordance with the law, the person concerned or a close relative retains the paper or electronic document and applies to a designated body for registration. The receiving body conducts a formal review of the document and, upon approval, digitizes it and uploads it to a nationally unified living will management platform established under government leadership. The platform stores the document in encrypted form and assigns it a unique registration number. It then synchronizes the information with the health-insurance information system and with hospital information systems. In this way, verifiable information that is valid nationwide is created, and—subject to strict data-protection and authorization rules—medical institutions are granted the right to directly consult a patient's living will under specified conditions, thereby reconciling authenticity, authoritativeness, and accessibility.

Admittedly, a fully unified national platform may face technological and financial constraints, particularly uneven digital infrastructure in rural areas and interoperability barriers between the national healthcare security information system and heterogeneous hospital information systems. To address implementation hurdles during the transition period, interim measures can be considered. For example, patients could be encouraged to carry wallet cards linked to a verified living will record to improve emergency accessibility in low-connectivity settings; in addition, provincial registries could be established first, using regional pilots to accumulate operational experience and gradually advance interprovincial data sharing, ultimately moving toward a nationally unified system. Such transitional arrangements can deliver practical access in the short term while laying the groundwork for the full build-out of a national platform.

#### 4.2.2 Improving the revocation mechanism for living wills

In practice, there is no consensus among countries and regions on the legislative models governing revocation of living wills, and the main divergences focus on two issues: first, the level of capacity required of the declarant at the time of revocation, that is, whether revocation must be effected personally by a declarant who possesses the requisite capacity; and second, whether revocation must take a prescribed formal form. Accordingly, two broad positions can be distinguished: a minority of states require that a living will be revoked in written form, whereas most do not mandate a written format; for example, both the US. UHCDA and the German Civil Code explicitly provide that a living will may be revoked in any form.

**Figure 2:**
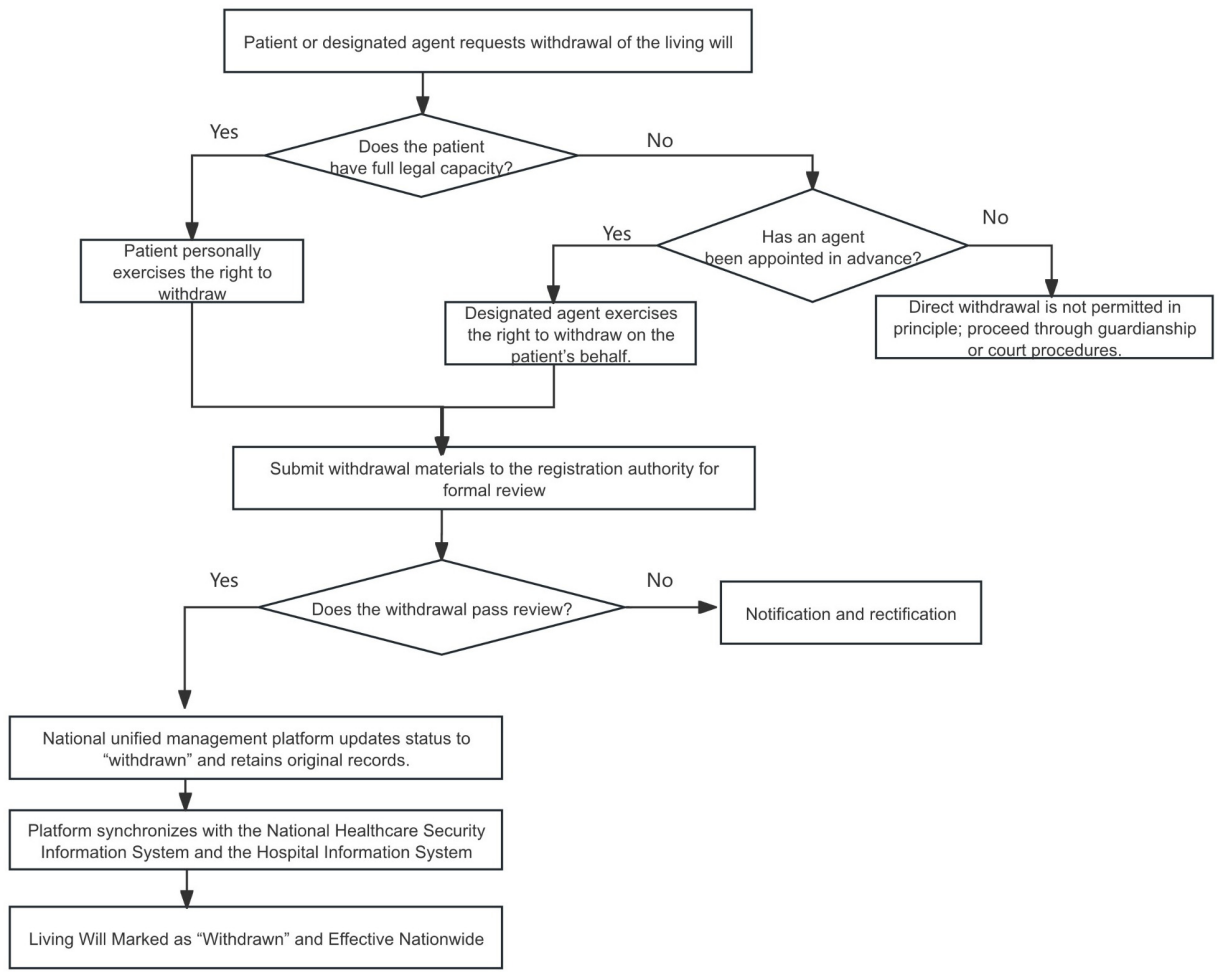
Withdrawal process of living wills. This flowchart depicts the procedure for amending or revoking a previously executed living will: initiating a withdrawal request by the declarant or an authorized surrogate, verifying identity and decision-making capacity, updating or canceling the registration record in the unified management system, and, where appropriate, notifying relevant medical institutions so that subsequent treatment decisions no longer rely on the revoked living will.

We argue that the revocation mechanism for living wills should, from the perspectives of protecting the right to life, reducing potential legal risks and disputes, and ensuring practicability, establish clear rules regarding both capacity and form (Figure 2). First, with respect to capacity, the default rule should be that only a patient with full civil capacity may personally revoke a living will. Where, for objective reasons, the patient cannot act in person, he or she may, by agreement, prospectively authorize a designated third party to exercise the right of revocation on his or her behalf. Second, with respect to form, only moderate formal requirements should be imposed. Comparative practice indicates that overly proceduralized safeguards may create administrative bottlenecks and delays, thereby impeding real-world operation even where the legal basis for living wills is formally recognized (45). Accordingly, revocation may be effected by a written statement, a notarial deed, or other reliable evidence sufficient to demonstrate an intention to revoke, so that its authenticity can be verified in the event of a dispute. At the same time, the procedure should not be unduly burdensome, nor must it mirror the form originally used to execute the living will: whether the will was made by notarization, written witnessing, or audio-visual witnessing, the declarant should be allowed to revoke it using any of these forms.

### 4.3 Long-term: promoting social acceptance through a locally grounded interpretation of the value of living wills

As Rousseau observed, “the ultimate law of all laws is engraved neither on marble nor on bronze, but in the hearts of every citizen” The implementation of any legal system must rest on public recognition and acceptance; otherwise, its effectiveness will be substantially diminished (46), and the establishment of a living will system is no exception. When elucidating the value foundations of the living will system, it is essential to consciously situate it within China's indigenous traditional value system and to reinterpret it in that light.

Although Confucian culture in everyday life tends to avoid explicit discussion of death, it nevertheless preserves intellectual traditions that place great importance on the end of life, notably the ideals of *shanzhong* (“a good death”) and *shenzhong zhuiyuan* (“paying careful attention to funerary rites and remembering the ancestors even when long gone”). In traditional society, arranging posthumous affairs, making inheritance wills, and choosing burial sites were essentially forms of advance planning for the final stage of life. Accordingly, in the context of modern medicine, the living will can be understood as an institutionalized expression of the ideal of a good death and as a proactive yet prudent arrangement by individuals concerning their later life and the manner of their dying. At the same time, the Confucian classics repeatedly emphasize that one should “follow the intentions of one's parents” (47). Filial piety is expressed not only in providing physical care to one's parents and sustaining their lives, but, more fundamentally, in respecting and fulfilling their genuine wishes. When parents, while still mentally competent, clearly set out their preferences regarding end-of-life treatment in a living will, and their children in practice respect and implement these wishes so as to prevent them from dying amid futile medical interventions and intense suffering, this in substance constitutes a higher form of filial piety. In addition, Chinese traditional medicine emphasizes that clinical decision-making should follow the natural course and the underlying logic of illness, rather than forcing interventions against it (48). From this perspective, a physician's implementation of a living will does not amount to abandoning professional duties. Instead, it can be understood as an ethical commitment to respecting the natural course of life and avoiding unwarranted or futile treatment. Applied to the practice of living wills, this line of thought can serve as a culturally grounded resource for helping clinicians navigate and ease ethical conflicts between beneficence and respect for patient autonomy.

To make this reinterpretation workable in clinical practice, we recommend introducing family conferences and embedding them in routine advance care planning before a crisis occurs. For example, healthcare institutions could arrange a family conference involving the patient (where possible), key family members, the attending clinician, and a trained facilitator (e.g., a social worker or a bioethicist). Such conferences can align the family's understanding of filial piety with the patient's expressed end-of-life directives, clarify prognosis and medical futility, and ease physicians' moral distress through dialogue, thereby making the above cultural reinterpretation workable in practice.

## 5 Conclusion

The emergence, consolidation, and progressive refinement of any legal system necessarily unfold through a gradual process, and this is especially true of the regime governing living wills, which directly implicates natural persons' lives, safety, and human dignity. This article reviews more than two decades of the evolution of living wills in mainland China and shows that the regime has shifted from an initial phase of civil-society advocacy to a phase of local legislative practice, while unified legislation at the national level remains absent. In the face of an increasingly pronounced trend of population aging, the development of a coherent national legislative framework in this field has become a matter of urgency. As a representative example of local legislative practice in this area, the Shenzhen Special Economic Zone Medical Regulations provide an explicit normative basis for living wills for the first time, making it of considerable value as a practical legislative experiment. Drawing on the practice of the Shenzhen Regulations, this article examines the deficiencies and challenges in the current legislative design and implementation of living wills, and on this basis offers systematic recommendations for improvement from three dimensions: clarification of key legal concepts, development of supporting mechanisms, and reinterpretation of the normative foundations of the regime. It further offers a detailed analysis of key issues such as the parties eligible to execute living wills, the conditions under which they take effect, the legal liability, and the mechanisms for registration and revocation, with a view to providing useful reference for China and for other countries that are still in the early stages of establishing living will systems.
