# Supplementary material for: The living will system in mainland China: legislative status quo, dilemmas and prospects
Source: Front Public Health. 2026 Feb 27;14:1767356. doi: 10.3389/fpubh.2026.1767356 (PMC12983372; doi:10.3389/fpubh.2026.1767356)
Supplement: Supplementary file 1 [file Table_1.docx]

Supplementary Material

This supplementary material includes Supplementary Table 1.

Supplementary Table 1.Major policy and legislative developments on living wills in China

| Major documents/events | Year | Issuing body | Main content related to living wills |
| --- | --- | --- | --- |
| Several Opinions on Promoting the Development of the Health Service Industry | 2013 | State Council of the People’s Republic of China | Expanding the supply of health services to meet diverse needs, and calling for accelerated development of health and elderly care services as well as the advancement of rehabilitation nursing, hospice and other related services. |
| Legislative proposal | 2013 | Gu Jin, deputy to the National People’s Congress | Drawing on relevant international experience, establishing a “living will registration center” and, through appropriate administrative laws or regulations, conferring the necessary legal effect on individually completed living wills. |
| Outline of the “Healthy China 2030” Plan | 2016 | Central Committee of the Communist Party of China and the State Council | Proposing that “attention should be paid to the entire life course so as to provide comprehensive health services and health security from the fetal stage to the end of life and to safeguard people’s health in an all-round way,” and advancing a series of major measures such as “active ageing,” including “providing integrated health and elderly care services with palliative care for older persons.” |
| Guiding Opinions on Establishing and Improving the Elderly Health Service System | 2019 | National Health Commission, National Development and Reform Commission and six other ministries and commissions | Providing palliative care services in accordance with the principles of patients’ full informed consent and voluntary choice, and establishing and improving multidisciplinary service models for palliative and hospice care so as to offer pain and symptom control, comfort care and related services for patients in the terminal stage of disease, as well as psychological support and humanistic care for patients and their families. |
| Civil Code | 2020 | National People’s Congress | Article 1002: A natural person enjoys the right to life. The safety and dignity of a natural person’s life are protected by law. No organization or individual may infringe upon another person’s right to life. |
| Legislative proposal | 2021 | Huang Gairong, member of the National Committee of the Chinese People’s Political Consultative Conference | Promoting living wills, namely written instruments signed while a person is mentally competent to express in advance his or her treatment preferences at the end of life. |
| Shenzhen Special Economic Zone Medical Regulations | 2023 | Shenzhen Municipal People’s Congress | Where a medical institution receives a living will provided by the patient or his or her close relatives that meets the following conditions, it shall, when implementing medical measures at the terminal stage of an incurable illness or at the end of life, respect the wishes expressed in the patient’s living will:  (1) The living will contains clear statements on whether to undertake or forgo traumatic resuscitative measures (intubation, cardiopulmonary resuscitation, etc.), whether to use life-support systems, and whether to continue treatment for the primary disease, etc.  (2) The living will has been notarized or witnessed by at least two witnesses, and the witnesses are not medical or health personnel involved in the patient’s treatment;  (3) The living will is made in written form or by audio or video recording; where it is not notarized and is made in written form, it shall be signed and dated by the testator and the witnesses, and where it is made by audio or video recording, the names or images of the testator and the witnesses as well as the time shall be recorded. |
| Legislative proposal | 2023 | Jiangsu Provincial Committee of the China Democratic National Construction Association | Drawing on Shenzhen’s legislative experience and incorporating legislation on living wills into the legislative agenda of the Jiangsu Provincial People’s Congress. |
| Legislative proposal | 2024 | Li Xiumei, member of the Zhejiang Provincial Committee of the Chinese People’s Political Consultative Conference | On the basis of reviewing and drawing on the experience and practices of other provinces and cities, relevant departments should be actively coordinated to explore legislative work on living wills and, at an appropriate time, advance the legalization of living wills. |
